# Investigation of lipid metabolism dysregulation and the effects on immune microenvironments in pan-cancer using multiple omics data

**DOI:** 10.1186/s12859-019-2734-4

**Published:** 2019-05-01

**Authors:** Yang Hao, Daixi Li, Yong Xu, Jian Ouyang, Yongkun Wang, Yuqi Zhang, Baoguo Li, Lu Xie, Guangrong Qin

**Affiliations:** 1 School of Medical Instrument and Food Engineering, University of Shanghai for Science and Technology, Shanghai, 200093 China; 2 Shanghai Center for Bioinformation Technology, Shanghai Academy of Science and Technology, Shanghai, 201203 China; 3 Institute for Systems Biology, Seattle, WA 98109 USA

**Keywords:** Lipid metabolism, Tumor immune micro-environment, Pan-cancer, Multiple omics analysis

## Abstract

**Background:**

Lipid metabolism reprogramming is a hallmark for tumor which contributes to tumorigenesis and progression, but the commonality and difference of lipid metabolism among pan-cancer is not fully investigated. Increasing evidences suggest that the alterations in tumor metabolism, including metabolite abundance and accumulation of metabolic products, lead to local immunosuppression in the tumor microenvironment. An integrated analysis of lipid metabolism in cancers from different tissues using multiple omics data may provide novel insight into the understanding of tumorigenesis and progression.

**Results:**

Through systematic analysis of the multiple omics data from TCGA, we found that the most-widely altered lipid metabolism pathways in pan-cancer are fatty acid metabolism, arachidonic acid metabolism, cholesterol metabolism and PPAR signaling. Gene expression profiles of fatty acid metabolism show commonalities across pan-cancer, while the alteration in cholesterol metabolism and arachidonic acid metabolism differ with tissue origin, suggesting tissue specific lipid metabolism features in different tumor types. An integrated analysis of gene expression, DNA methylation and mutations revealed factors that regulate gene expression, including the differentially methylated sites and mutations of the lipid genes, as well as mutation and differential expression of the up-stream transcription factors for the lipid metabolism pathways. Correlation analysis of the proportion of immune cells in the tumor microenvironment and the expression of lipid metabolism genes revealed immune-related differentially expressed lipid metabolic genes, indicating the potential crosstalk between lipid metabolism and immune response. Genes related to lipid metabolism and immune response that are associated with poor prognosis were discovered including HMGCS2, GPX2 and CD36, which may provide clues for tumor biomarkers or therapeutic targets.

**Conclusions:**

Our study provides an integrated analysis of lipid metabolism in pan-cancer, highlights the perturbation of key metabolism processes in tumorigenesis and clarificates the regulation mechanism of abnormal lipid metabolism and effects of lipid metabolism on tumor immune microenvironment. This study also provides new clues for biomarkers or therapeutic targets of lipid metabolism in tumors.

**Electronic supplementary material:**

The online version of this article (10.1186/s12859-019-2734-4) contains supplementary material, which is available to authorized users.

## Introduction

Reprogramming of cellular metabolism is a well-established hallmark of tumor that has attracted increasing attention in the recent years [1]. Among the most important biology components, lipids have many key biological functions, i.e. acting as cell membrane components, serving as energy storage sources and participating in cell signaling [2, 3]. The perturbations of lipid metabolism in cancer cells may alter cellular function dramatically. Metabolic reprograming also provides critical information for clinical oncology, and the recent study from the pan-cancer study showed a clinical relevance of metabolic expression subtypes in human cancers [4]. Over the past few decades, many important discoveries regarding genes that regulate lipid synthesis and degradation have led to the current understanding of the complex biochemical reactions in the metabolism transduction pathways. However, different types of lipids may have different features in different cancer types. Up to now, there is still no comprehensive study of the lipid metabolism in pan-cancer.

Furthermore, increasing evidences suggest that the alterations in tumor metabolism can also contribute to the inhibition of the antitumor response. Immunosuppression in the tumor microenvironment (TME) is suggested to be based on the mutual metabolic requirements of immune cells and tumor cells [5]. Abnormal lipid metabolism occurres not only in tumor cells but also in TME. A previous study demonstrated the deletion of 5-Lipoxygenase in the TME promoted lung cancer progression and metastasis through regulating T Cell recruitment [6]. Thus, the investigation of the lipid metabolism features as well as the crosstalk between the lipid metabolism and the tumor immune microenvironment could help us to understand the tumorigenesis in the solid tumors.

In the past 10 years, multiple omics data for different tissue-origin tumors have been generated in The Cancer Genome Atlas (TCGA) project, which provides a rich resource for understanding tumor features. According to the tumor origin, tumors are classified into pan-gastrointestinal, pan-gynecological, pan-kidney and pan-squamous tumors etc. However, recent studies have shown both commonalities and differences in genetic mutations [7] exist among different tissue-origin tumors. The integrative analysis of gene expression, mutation and DNA methylation could not only draw a landscape of the alteration of lipid metabolism pathways, but also give clues to the regulation of lipid metabolism. A comprehensive analysis of lipid metabolism in different tissue specific cancers using multiple omics data may provide novel insights in tumorigenesis and progression. At the same time, tools or algorithms were developed in characterizing cell composition of complex tissues from their gene expression profiles, especially for the immune cell compositions, such as CIBERSORT [8] and TIMER [9]. The data resources and tools may help us to study the lipid metabolism features and their relations to the immune microenvironments. In the present work, we explored the key altered lipid metabolism pathways in pan-cancer, and investigated the regulation of these pathways through the integration of mutation, DNA methylation and transcription factors. We also analyzed the crosstalk of the key altered lipid pathways with other oncogenic pathways, as well as the correlation of the expression of lipid metabolism genes with the proportions of immune cells in the TME and prognostic effects of genes in the lipid metabolism pathways.

## Results

### Key altered lipid metabolism pathways in pan-cancer

**Fig. 1:**
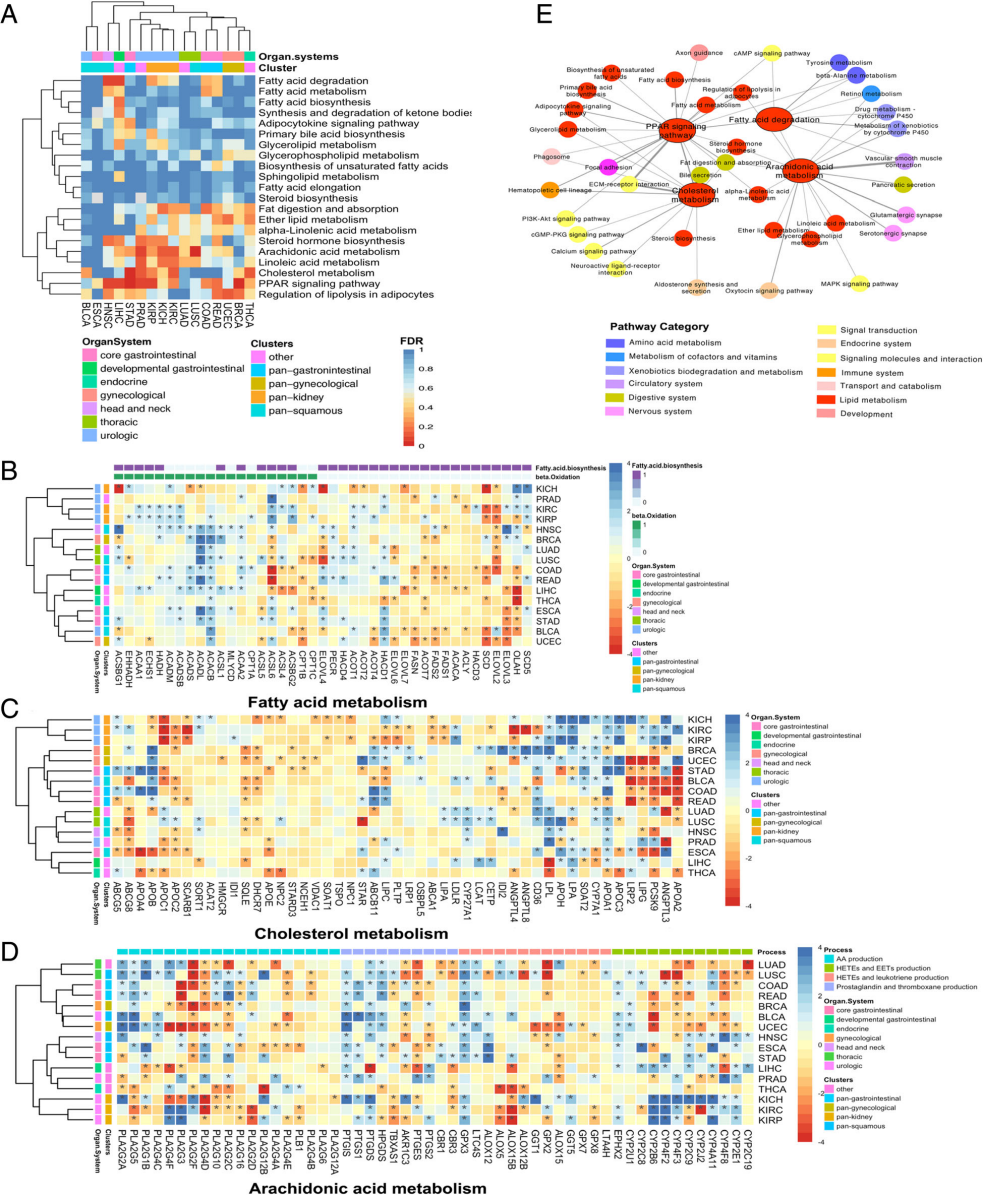
Alterations in the lipid metabolism pathways and the crosstalk with other pathways. **a** Heatmap of the enrichment significance score (colored by FDR) of lipid metabolism pathways from the KEGG pathways in pan-cancer. **b**, (**c)** and (**d**) Heatmaps of differentially expressed genes (blue: down-regulation; red: up-regulation) related fatty acid metabolism, cholesterol metabolism and arachidonic acid metabolism in pan-cancer. Significantly differentially expressed genes were highlighted with * (FDR < 0.05). **e** The network of lipid metabolism pathways with other pathway with shared differentially expressed genes. Pathways were colored by their pathway categories

Sixteen solid tumor types were selected in the present study with a criterion of the number of para-tumor samples in RNA level over ten samples, namely BLCA, BRCA, COAD, ESCA, HNSC, KICH, KIRC, KIRP, LIHC, LUAD, LUSC, PARD, READ, STAD, THCA and UCEC. Differentially expressed genes were detected in each tumor type comparing the tumor samples with the paired adjacent normal tissue samples respectively using edgR [10]. Pathway enrichment analysis (Fisher exact test, Benjamini & Hochberg adjustment) was performed according to the differentially expressed genes with the background pathway database KEGG. From the twenty-one selected lipid-metabolism-related pathways (Additional file 1), the result shows that the most widely and significantly altered lipid metabolism or related signaling pathways in pan-cancer are PPAR signaling, fatty acid degradation, arachidonic acid metabolism pathway and cholesterol metabolism pathway (Fig. 1a). Unsupervised clustering of the pan-cancer by the pathway enrichment false discover rate (FDR), shows that similar lipid metabolism features are shared among the similar tissue origin tumors. For example, the pan-kidney cancers or tumors from urologic organism show similar lipid metabolism pattern including the significantly altered cholesterol metabolism, PPAR signaling pathway and arachidonic acid metabolism (Fig. 1a). The lung cancers such as LUAD and LUSC also show similar lipid metabolism pattern.

We next analyzed the gene expression landscape of the key altered lipid metabolism pathways. It can be observed that the fatty acid metabolism process shows common features among various tumor types. Unsupervised clustering of genes expression alterations in fatty acid metabolism pathway reveals that genes which regulate the beta oxidation process in mitochondrion are dominantly down-regulated in most of the cancer types. Inversely, the genes specific for fatty acid biosynthesis were dominantly up-regulated in tumor samples compared to adjacent normal samples (Fig. 1b), suggesting an accumulation of fatty acid in tumor cells. The gene expression in the cholesterol metabolism pathway showed tumor specificity. Clustering pan-cancer based on the cholesterol metabolism pathway, three sub-groups can be classified, including the pan-kidney cancer (KICH, KIRC and KIRP) group, the pan-gynecological (BRCA, UCEC) and pan-gastrointestinal cancers (STAD, COAD and READ) group, and the third group including LUAD, LUSC, HNSC, PRAD, ESCA, LIHC and THCA (Fig. 1c). The alteration of arachidonic acid metabolism pathway also shows strong tissue specificity in different cancers. The arachidonic acid metabolism pathway can be divided into four sub-processes, including the generation of arachidonic acid catalyzed by phospholipase A2 (PLA2), and the production of downstream products by various enzymes, including the production of prostaglandins by the catalysis of cyclooxygenases (COXs), the production of leukotriene and hydroperoxyeicosatetraenoic acids (HETEs) by lipoxygenases (LOXs) and the production of several HETEs and epoxyeicosatrienoic acids (EETs) by cytochrome P450 (CYP450) epoxygenases enzymes [11]. Our results revealed that in the arachidonic-acid-producing process, the key regulation gene PLA2G10 was significantly up-regulated in KIRC, KICH, THCA, BRCA, UCEC and LUAD, while down-regulated in COAD, READ and LUSC (Fig. 1d). Regarding the effects of PLA2, we found that in LIHC, only significantly up-regulated genes were observed in arachidonic acid producing process, suggesting the over-production of arachidonic acid in LIHC. For the production of HETEs and EETs, CYP450 family genes play important roles. In HNSC, ESCA, STAD, LIHC and the pan-kidney tumors, CYP450 family genes were dominantly down-regulated, indicating the functional reduction of the HETEs and EETs production. These results reveal different types of lipids show different expression features in pan-cancer.

To further investigate the potential influence of lipid metabolism in cancer, we investigated the crosstalk between the four widely significantly altered pathways and other pathways by comparing the shared differentially expressed genes. We found that the alteration of the lipid metabolism pathways can affect metabolism, signaling transduction and immunity. Specifically, the alteration of fatty acid degradation is also companioned with other metabolism pathways such as amino acid metabolisms and xenobiotics, as well as the cAMP signaling pathway. The alteration of arachidonic acid metabolism can not only affect other metabolism process, but also accompanied the alteration of signaling pathway such as the MAPK signaling pathway. The differentially expressed genes in the cholesterol metabolism pathway can also modulate multiple signaling pathways such as PI3K-Akt signaling, calcium signaling, cGMP-PKG signaling pathway etc., impling its important role in signaling modulation. The alteration in the cholesterol metabolism pathway may also side with the ECM-receptor interaction and the immune systems (e.g. Hematopoietic cell lineage), revealing its role in cell attachment and immune-effects. As a key modulation signaling pathway, the PPAR signaling can modulate the fatty acid metabolism, arachidonic acid metabolism and cholesterol metabolism (Fig. 1e).

### The regulation of lipid metabolism pathways (methylation/ mutation/transcription)

**Fig. 2:**
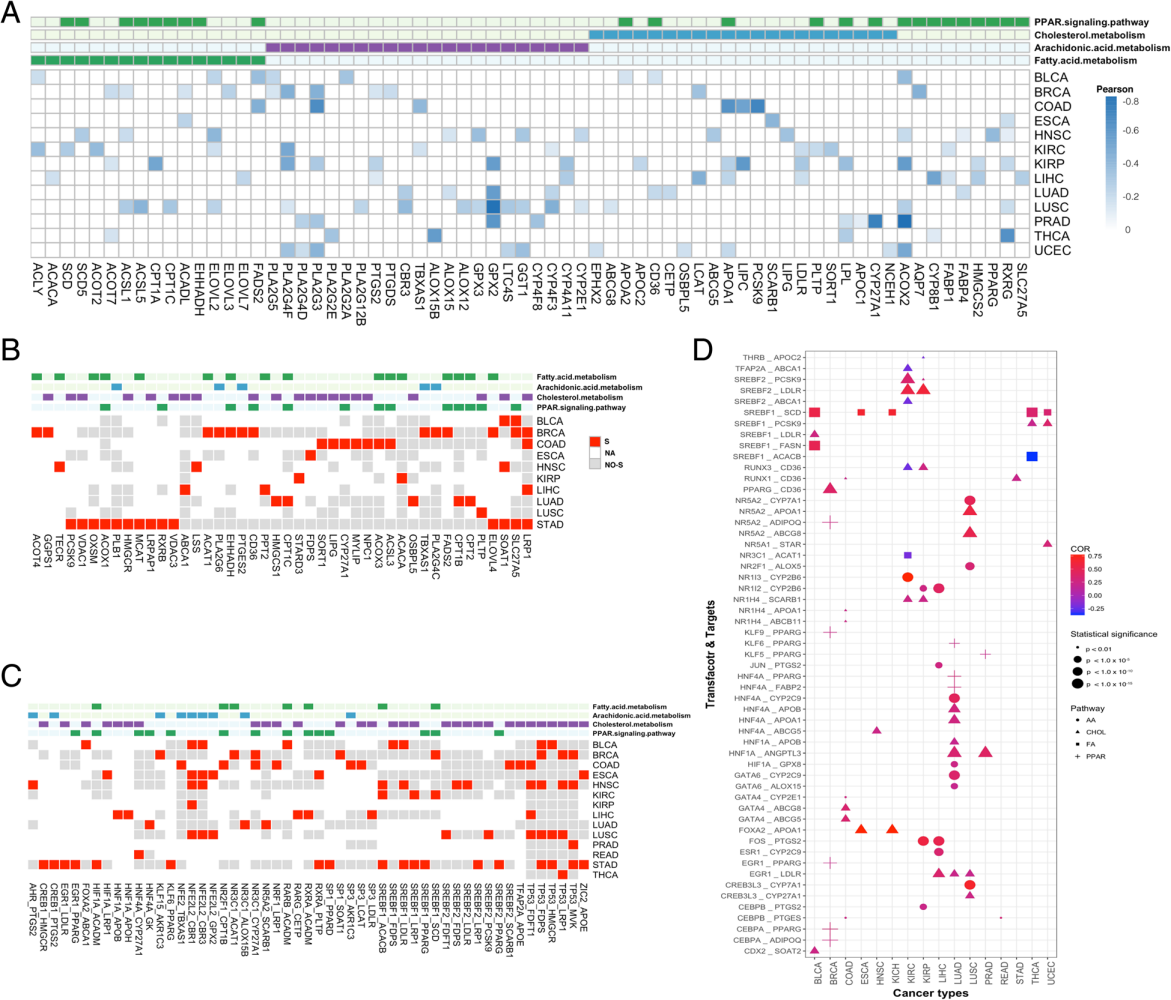
Multiple mechanisms contribute to dysregulation of lipid metabolism genes in cancer. **a** Pearson correlation estimation (*p* < 0.05) of the association between mRNA expression and DNA methylation in the promoter region across pan-cancer. **b** Gene mutation versus its mRNA expression (red color represents there is significant (wilcox test, *p* < 0.05) difference of the mRNA expression between the mutated group and the non-mutated group, while grey color represents that no significant difference observed between the mutated group and the non-mutated group. To perform statistic testing, the number of samples in either the mutated group or the non-mutated group should be greater or equal to three. The white color represents the gene is with less than three mutated samples.). **c** Transcription factor mutation versus targets expression (Color as the same as B). **d** Pearson correlation estimates of association between the expression of transcript factors and their target genes across pan-cancer. The circle size is proportional to the significance level of correlation results

To examine the molecular mechanisms underlying the transcriptional regulation of the lipid genes in cancer, we further integrated DNA methylation, somatic mutation and mRNA expression data. We mainly focused on how fatty acid metabolism, arachidonic acid metabolism, cholesterol metabolism and PPAR signaling pathway were altered in pan-cancers in multiple omics level. Firstly, we explored the effect of DNA methylation on gene expression for the lipid metabolism genes. Based on the same tissue samples, we detected differentially methylated sites comparing the DNA methylation profiles in tumor with the adjacent normal tissues. A former study has shown that abnormal DNA methylation in the promoter region has a more important regulatory effect on gene expression than non-promoter regions [12], and cis-regulation was dominated by the negative correlation between promoter methylation and gene expression [13]. We detected the differentially methylated CpG sites (q-value < 0.05, dbeta > 0.1) that mapped on the promoter regions and significantly negatively correlated with the differentially expressed genes. Averaging of differential methylation sites located on the same gene that were negatively correlated with the expression of the differentially expressed genes in the four pathways were shown in Fig. 2a. It can be observed that some of the differentially expressed lipid metabolism genes were regulated by DNA methylation. i.e., APOA1, which has anti-inflammatory and antioxidant properties [14], was reduced in gene expression associated with hyper-methylation of its promotor region in cholesterol metabolism pathway, which could accelerate tumor growth and metastasis primarily via modulation of innate and adaptive immune responses in tumors [15, 16]. PLA2 (PLA2G4F), which catalyzes the hydrolysis of membrane phospholipids to release arachidonic acid (AA), was up-regulated in BRCA and UCEC (pan-gynecological) accompanied with DNA hypo-methylation, inversely, down-regulated in KIRC and KIRP (pan-kidney) accompanied with hyper-methylation in the promoter regions, respectively. The differential methylation pattern supports the tissue specific expression landscape of lipid metabolism pathways.

Next, we identified somatic alternations that potentially regulated lipid metabolism. (See Additional file 2) depicted the lipid genes with mutation frequency of more than 2% and differently expressed in the four significantly altered lipid-metabolism pathways in pan-cancer. More high-frequency-mutated genes were observed that accompanied with dysregulated mRNA expression in UCEC, while fewer genes were observed in KICH, KIRC and KIRP. Notably, some genes with high frequency mutation also showed consistent gene differential expression patterns across pan-cancer. i.e., ACACB and RXRG were with high mutation frequency and down-expressed in most cancer types, including UCEC, LUAD, LUSC, COAD, BLCA and HNSC. As gene mutations commonly occurred in a part of the tumor samples, we further investigated whether the expression of genes was directly associated with mutations in tumor samples. Candidate lipid genes with mutations in at least three tumor samples were selected, grouped by gene mutations. Wilcox test was performed to detect the difference between mutanted and non-mutated groups (Fig. 2b). In BRCA, COAD and STAD, some mutated genes are significantly correlated with mRNA expression. Compared with mRNA expression profile, we found the over-expression of FADS2 and ELOVL4, and the low-expression of CD36 and LRP1 in BRCA were associated with the somatic mutations of the same genes. The over-expression of LIPG in COAD and PCSK9 in STAD, the low-expression of HMGCS1 in LUAD and ACACB in STAD may also be regulated by gene mutations.

Mutation and differential expression of transcription factors could also be critical factors for the regulation of lipid metabolism. In the present study, we selected transcription factor and target gene pairs from TRANSFAC [17] (Additional file 3). Firstly, we analyzed the effect of mutations of transcription factors on the expression of target lipid-related genes. The pan-cancer map of mutated transcription factors significantly associated (Wilcoxon signed-rank test) with targeted lipid genes expression is shown in Fig. 2c. The mutations of SREBF1, SREBF2 and TP53 show significant impacts on downstream targeted lipid genes, especially in the downstream target genes in cholesterol metabolism pathway. In addition, the mutation on SREBF1 was also associated with the expression of ACACB and SCD, which are important enzymes in fatty acid beta-oxidation and the synthesis of unsaturated fatty acids processes, respectively. In BRCA, HNSC, THCA and LUSC, TP53 mutation regulated the expression of LRP1, the up-regulation of LRP1 has been reported to be associated with the invasiveness of cancer cells by supporting ERK and inhibiting JNK signaling pathways [18, 19]. Secondly, we analyzed the Pearson correlation of the expression levels between the transcription factors and downstream targets in tumor samples. Figure 2d shows the differentially expressed transcription factors which are significantly associated with the expression of the targeted lipid-metabolism genes (absolute Pearson correlation > 0.2, *p*-value < 0.01). The expression of SREBF1 is closely associated with targeted lipid-metabolism genes in fatty acid metabolism and cholesterol metabolism pathway. It indicates that the up-regulation of SREBF1 promoted the de novo synthesis of fatty acids associated with the upregulation of ACLY, FASN and SCD, increased cholesterol uptake into hepatocytes associated with the upregulation of PCKS9, modulated the fatty acid oxidation in mitochondria associated with the down-regulation of ACACB in most cancers (Fig. 2d, Additional file 4). Similarly, the abnormal expression of SREBF2 also significantly affected the imbalance of cholesterol metabolism and arachidonic acid metabolism.

Strikingly, based on the integration analysis of multi-omics data, we found that many regulatory factors lead to the same result. In COAD, the significant up-regulation of TBXAS1 expression is affected by hypo-methylation of the DNA promoter region and associated with mutation of the upstream transcription factor NFE2. In LIHC, the significant downregulation of LDLR expression may be modulated by the hyper-methylation of the DNA promoter region, the mutation of the upstream transcription factor SP3, and the down-regulation of the transcription factor EGR1. These results provide a more comprehensive and illustrative mechanism of tumor lipid metabolism abnormalities.

### The correlation between lipid metabolism and the immune cells in tumor micro-environment

**Fig. 3:**
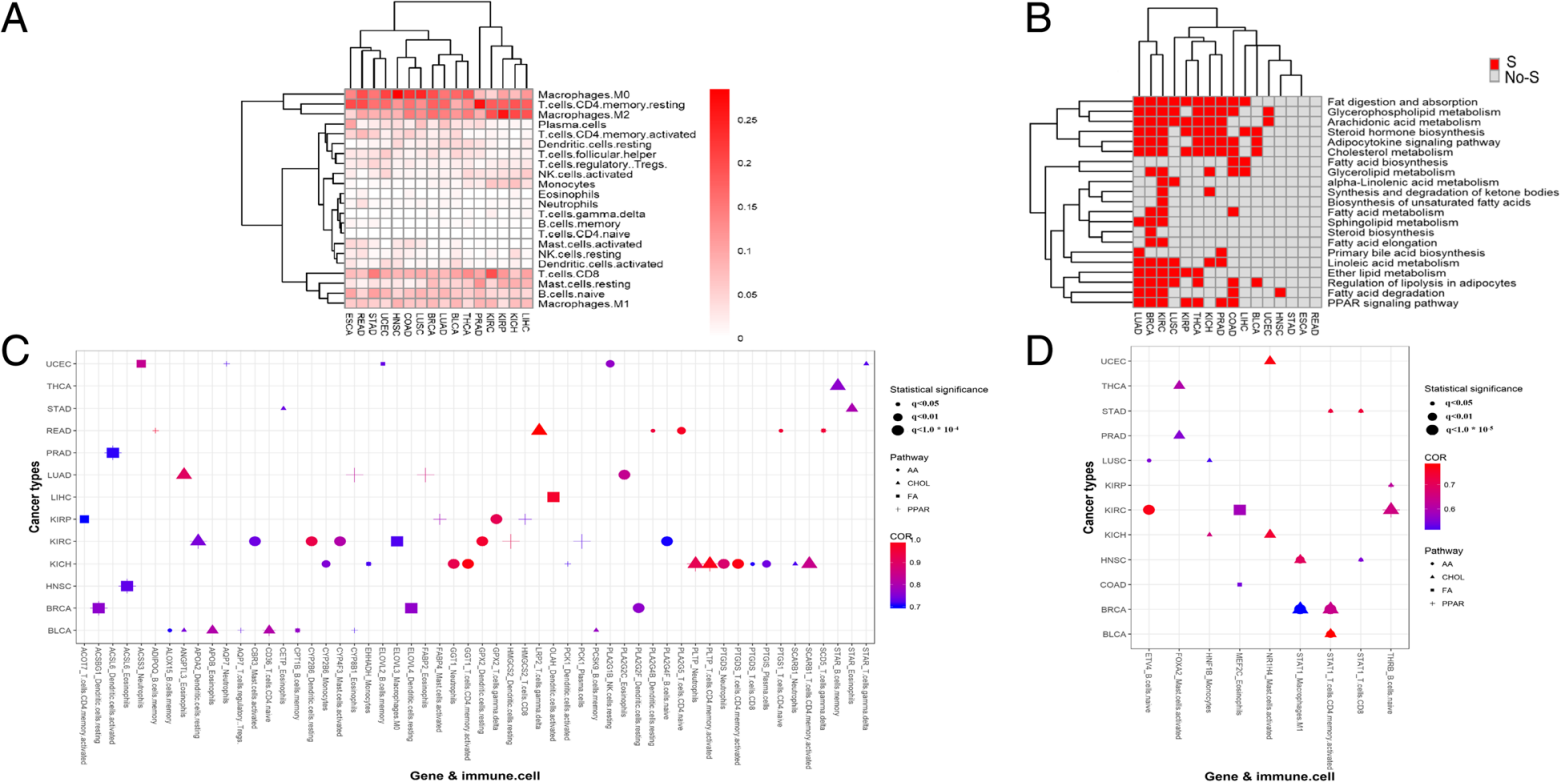
The correlation between lipid metabolism and the immune cells in tumor micro-environment**. a** Heatmap of the average proportion of the immune cells (colored by proportion) in pan-cancer. **b** Heatmap of enrichment significance of lipid metabolism related pathways from KEGG pathways for the differentially expressed lipid metabolism genes which are significantly related immune in pan-cancer (colored by FDR, red represented significant enrichment (FDR < 0.05) and grey represented the non-significant enrichment (FDR > = 0.05)). **c** Pearson correlation between the expression of differentially expressed lipid metabolism related genes and the proportion of immune cell in pan-cancer. **d** Pearson correlation between the expression of differentially expressed transcription factors and the proportion of immune cell for pan-cancer

The proportion of immune cells in the tumor microenvironments of the sixteen tumors were estimated using CIBERSORT [20]. We found that the Macrophages M0, T cell CD4 memory resting and Macrophages M2 were among the highest-proportion immune cells across all the sixteen tumor types. While, Eosinophils, Neutrophils, T cells gamma delta, B cells memory and T cells CD4 naive are among the lowest-proportion immune cells (Fig. 3a). Unsupervised clustering of the immune cell proportions can classify the pan-cancers into three sub-groups. The pan-kidney cancer (KICH, KIRC and KIRP), LIHC and PRAD were clustered in to one sub-group featured with high proportion of T cells CD4 memory resting. HNSC, COAD, LUSC, BRCA, LUAD, BLCA and THCA were clustered into another subgroup featured with high proportion of Macrophages M0, and the other cancer types including pan-gastrointestinal (ESCA, READ and STAD) fall into the third group. To further explore the relation between lipid metabolism and the immuno-microenvironment, Pearson correlation between expression values of the lipid genes and the immune cell proportions were carried out. Genes that are significantly correlated with at least one of the 22 immune cells and significantly differentially expressed were further used to perform pathway enrichment analysis. The enrichment score (FDR) of the 21 selected lipid metabolism pathways were shown in Fig. 3b. It can be observed that the differentially expressed lipid metabolism genes which were significantly correlated with tumor immune environments were enriched in the cholesterol metabolism pathway and arachidonic acid pathway. We further selected the most significantly correlated genes with absolute correlation coefficient greater than or equal to 0.7 in the four significantly altered lipid metabolism pathways (Additional file 5). The highly correlated lipid-metabolism genes with the immune cell proportions in the four pathways are shown in Fig. 3c, including ANGPTL3, PLTP and LRP2 in the cholesterol metabolism pathway, PLA2G5, CYP2B6 and GPX2 in the arachidonic acid metabolism pathway, HMGCS2 and FABP2 etc. in the PPAR signaling pathway, OLAH and SCD5 etc. in the fatty acid metabolism indicating their roles in altering the TME. We further assessed the correlation of the expression level of transcription factors which regulate lipid metabolism and the proportion of immune cells. Transcription factors associated with immune cells were selected with the thresholds of absolute correlation coefficient greater than 0.5 and FDR < 0.05 in at least two tumor types, as shown in Fig. 3d. Specifically, we found that the transcription factor STAT1, which is a key regulator for the cholesterol metabolism and arachidonic acid pathway, was significantly correlated with the proportion of T cells CD4 memory activated, T cells CD8 and Macrophages M1, implying its role in regulating the immune microenvironment by acting on lipid metabolism.

### Prognosis impacts of lipid metabolism

**Fig. 4:**
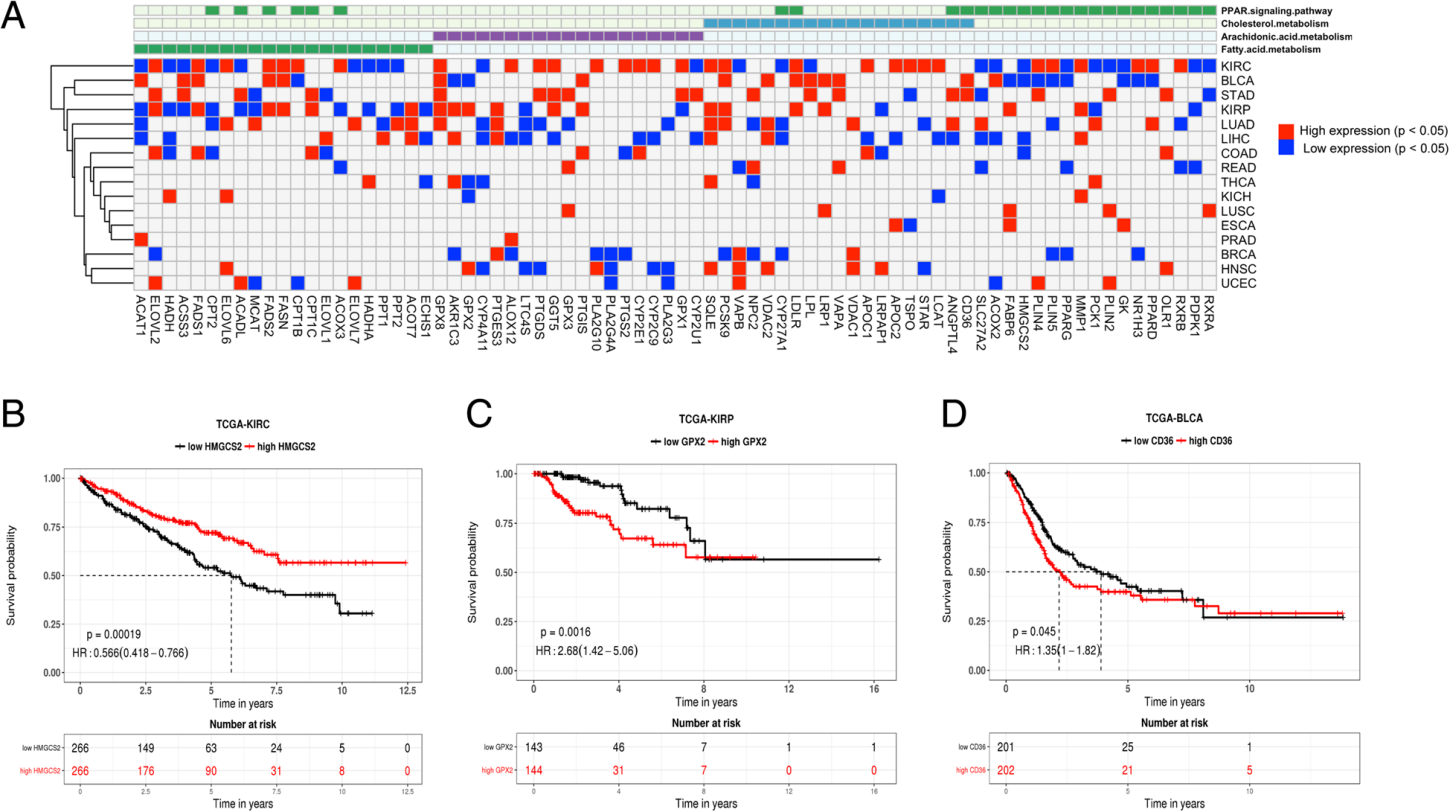
Prognosis impact of genes in key lipid metabolism pathways. **a** Genes in the four key altered lipid metabolism pathways which are associated with prognosis. Red represents high gene expression significantly reduces the patient’s overall survival time and blue represents low gene expression significantly reduces the patient’s overall survival time. **b**, (**c**) and (**d**) Kaplan-Meier survival curve for tumors stratified by high or low expression levels of **(b)** HMGCS2, (**C**) GPX2 and (**d**) CD36 (the high or low expression level groups were stratified by the median expression value). *P*-value indicates significance levels from the comparison of survival curves using the Log-rank test

To investigate the prognosis impact of lipid metabolism, especially genes in the four most widely significantly altered pathways, we classified samples from each of the sixteen cancer types into two groups according to the median value of the gene expression for each gene. Log-Rank test was performed to estimate the difference between the survival time of the two groups. The lipid-metabolism related genes with significantly prognosis impact were shown in Fig. 4a (*p*-value < 0.05). High-expression of FADS1, FADS2, FASN and ACOT7 in the fatty acid metabolism pathway, GPX8, PTGES3 and PTGIS in the arachidonic acid metabolism pathway, SQLE, VDAC1, CD36, LDLR, LRP1 and VAPA in the cholesterol metabolism pathway and MMP1, OLR1 in the PPAR signaling pathway significantly reduced the overall survival of patients. While down-regulation of genes such as CYP4A11, PLA2G4A, PLA2G3, LTC4S, CYP27A1, HMGCS2 and PDPK1 reduced patient overall survival time in most tumor types. Taking the differential expression between tumor and adjacent normal samples into consideration, it can be observed that the lipid-metabolism related genes, such as MMP1, HMGCS2 and AKR1C3, may not only be diagnosis markers for tumors but also may act as prognosis biomarkers in different tumor samples. These results indicate that abnormal lipid metabolism in tumors may play crucial roles in patient survival.

Furthermore, we found three lipid-metabolism related genes HMGCS2, GPX2 and CD36 associated with the tumor immune microenvironment were significantly correlated with prognosis (Fig. 4b, c and d). Low expression of HMGCS2 was associated with poorer survival time in KIRC. High expressions of GPX2 and CD36 were associated with poorer survival in KIRP and BLCA, respectively. These results may provide important biomarkers for the potential prognosis and treatment of cancers from an immune aspect.

## Discussion

This comprehensive integrated analysis of pan-cancer enhances our understanding of the lipid metabolism dysregulation molecular events relevant to multiple cancer types. Our analysis highlights the most widely significantly perturbed lipid metabolic pathways in cancers, including PPAR signaling, fatty acid degradation, arachidonic acid metabolism pathway and cholesterol metabolism pathway. The synthesis and degradation of fatty acids which provide the material and energy base for tumors, the metabolism of arachidonic acid produces different inflammatory cytokines, the cholesterol metabolism may influence many signaling pathways and the PPAR signaling pathway can directly regulate other lipid metabolism pathways. Based on the analysis of gene expression profiling for multiple tumors, the metabolic processes of fatty acids appeared to be altered in a more consistent way in most tumors, including the attenuation of fatty acid beta oxidation process in mitochondrion and enhancement of fatty acid de novo biosynthesis in tumor samples, suggesting an accumulation of fatty acid in tumor cells, which support the synthesis of cell membranes and cell division requirements. The alteration of arachidonic acid metabolism pathway and cholesterol metabolism show strong tissue specificity, which may reflect the different inflammatory or immune environments in different types of tumors. By exploring the regulation of gene expression in lipid metabolism, the integrated analysis of DNA methylation, mutation and the expression of transcription factors provide further evidence for the lipid metabolism. Different expression pattern in different lipids metabolism pathways may be influenced by their micro-environments. Further correlation analysis between the gene expression of lipid-metabolism genes and the immune cell proportions in the tumor immune microenvironments further provides clues for the relation between lipid metabolism and the immune microenvironments. For example, the expression of transcription factor STAT1 which modulate the cholesterol metabolism and arachidonic acid pathway, was correlated with several types of immune cells in the tumor immune microenvironment. Following prognosis analysis revealed that several lipid-metabolism genes may not only be diagnosis biomarkers for tumors but may also be prognosis biomarkers, such as MMP1 and HMGCS2. It has been reported that MMP1 overexpression has an important role in promoting tumor cell invasion [21] and HMGCS2 silence promoted tumor cells metastatic via Epithelial-Mesenchymal Transition (EMT) process and the activation of ERK/c-Jun signaling pathway [22]. We also revealed lipid metabolism biomarkers that were closely correlated with the tumor immune microenvironment, such as CD36. Former research investigated that the increased expression of CD36 promotes tumor metastasis, and mediates a pro-apoptotic effect in ovarian tumor cells [23]. Genetic depletion of the fatty acid translocase CD36 inhibits the induction of immunosuppressive function in tumor-infiltrating Myeloid-derived suppressor cells (MDSC) and results in a CD8^+^ T cell-dependent delay in tumor growth [24].

Our results provide a comprehensive analysis of lipid metabolism in tumors and explored the association between lipid metabolism and immune environments, which could further provide clues for lipid-metabolism based diagnosis biomarker discovery and prognosis biomarker development. There are still some limitations in the present study. The correlation analysis between the gene expression of the lipid metabolism genes and the proportions of immune cells implys their association, however, generating such data is not enough to capture detailed interaction. Whether abnormal lipid metabolism is a driving force for immune microenvironment formation or whether abnormal immune microenvironment can lead to abnormal lipid metabolism still need to be validated experimentally.

## Conclusion

Based on multi-omics data of pan-cancer, we found widely alterations of fatty acids, arachidonic acid and cholesterol metabolism and PPAR signaling in different tumors, and similar lipid metabolism features are shared among the similar tissue origin tumors. In the process of studying the mechanism of abnormal regulation of expression profile, we correlated possible causes of metabolic disorders of lipids in tumors from several aspects: somatic mutation, DNA methylation abnormality and regulation of transcription factors. This would contribute to providing clues to support the further molecular regulatory experiments. Our analysis revealed potential correlation between lipid metabolism and immune response. In addition, we also found genes related to lipid metabolism and immune response that are associated with poor prognosis. It is of great significance to molecular targeted therapy for tumors and development of new anti-cancer drugs. In conclusion, integrated analytic approaches have been applied to multiple data platforms from a large set of clinically annotated multiply tumor cases to provide a better understanding of lipid dysregulation molecular targets that may lead to improved therapeutic strategies.

## Methods

### Data acquisition

Multiple omics data including gene expression data normalized by RSEM from Illumina HiSeq RNASeqV2, DNA methylation data from the Human Methylation450 assay, DNA mutation data and clinical data were downloaded from Broad GDAC Firehose [25]. Sixteen solid tumors with sample size of adjacent normal samples over 10 were selected in this study, including BLCA, BRCA, COAD, ESCA, HNSC, KICH, KIRC, KIRP, LIHC, LUAD, LUSC, PRAD, READ, STAD, THCA and UCEC. Gene expression data (Raw counts and FPKM) were downloaded from GDC Data Portal [26]. The HumanMethylation450 assay obtained as the DNA methylation status (β values) range from zero to one, with scores of “0” indicating no DNA methylation and scores of “1” indicating complete DNA methylation.

### Differential gene expression analysis

Genes were taken into consideration for differentially expressed gene calculation, with a minimum sample size with detection over six and over a quarter of the total sample size. Differential expressed genes between tumor and paired adjacent normal samples were detected using edgeR [10] using the gene expression data of raw counts. A threshold of FDR less than 0.01 and the absolute log2 fold-change greater than 1 were used for defining differentially expressed genes.

### Pathway enrichment analysis

Pathway enrichment analysis was performed using fisher exact test followed by Benjamini & Hochberg test based on KEGG pathways using the differentially expressed genes for each tumor. Pathways with FDR less than 0.05 were considered to be significantly enriched pathways. According to KEGG pathway categories, lipid metabolism pathways were selected in the category of Metabolism and Organismal systems, as listed in Additional file 1. Pathway enrichment score for the lipid metabolism pathways were plotted in Fig. 1a using ‘pheatmap’ in R package according to the FDRs.

### Differential DNA methylation analysis

To study the differentially methylated sites between tumor and adjacent normal tissue, we used the Bioconductor tool, minfi [27] for differential methylation analysis. Probes which had “NA”-masked data points or located on sex chromosomes were eliminated. For a CpG site to be considered differently methylated, the difference in the median value between the tumor and the paired normal samples (dbeta) should be more than 0.1 and q value less than 0.05 were considered statistically significant.

### Integrated analysis of methylome and transcriptome

For analyzing the effect of methylation on gene expression, only CpG sites located in the promoter region (TSS1500, TSS200, and 5′ UTR) which showed differential methylation were taken into consideration. For specific gene in one cancer type, hypo-methylation in the gene promoter region and over-expression, or hyper-methylated in the promoter region and down-expression were considered as positively regulations [28]. Pearson correlation between gene expression and averaging of gene-associated probes was evaluated.

### Integrated analysis of somatic mutations and transcriptome

For a specific gene, the mutations on the gene may influence its expression. To investigate the effect of somatic mutations on gene expression, we grouped the tumor samples according to mutations for each gene, then Wilcoxon signed-rank test was used to identify the difference of gene expression between the mutated group and the non-mutated group. On the other hand, gene mutations in the transcription factors may also influence targets. The transcription factor and target gene pairs were extracted from TRANSFAC database [29]. To evaluate the effect of mutation in transcription factors to the expression of downstream targets, we classified samples according the mutation states of specific transcription factors. The Wilcox test was also used to identify the expression difference of the gene expression level of targets. For the above analysis, genes with mutations in at least three tumor samples were taken into consideration. *P*-value < 0.05 were considered statistically significant.

### Correlation analysis between transcription factors and targets

To investigate the effect of transcriptional regulation of lipid metabolism, we further assessed the correlation between the expression of the transcription factors and lipid metabolism genes. Correlations between transcription factors and targets were assessed using Pearson correlation. Significant correlations were considered as those pairs with *p*-value smaller than 0.05.

### Correlation analysis between tumor immune cell proportion and lipid metabolism specific gene expression

The relative abundance of 22 immune cell types in the sixteen cancer types was estimated using CIBERSORT [8, 20] based on the gene expression data from TCGA. To investigate the effect of lipid metabolism to immune microenvironment in tumors, we assessed the correlation between the expression of candidate lipid genes and transcription factors collected from the literature, KEGG and TRANSFAC databases to filter by the differentially expressed genes in the category of lipid metabolism (Additional file 6) and each tumor immune cells proportion in each tumor type using Pearson correlation followed by BH adjustment. Lipid-metabolism genes correlated with immune cell proportions were selected with the threshold of FDR smaller than 0.1. Pathway enrichment analysis of the lipid-metabolism genes correlated with the immune cells were performed using Fisher exact test followed by BH adjustment based on KEGG pathways.

### Prognosis analysis of differentially expressed genes

To investigate the prognostic impact of lipid metabolism, we performed survival analysis on the differently expressed genes related fatty acid metabolism, arachidonic acid metabolism, cholesterol metabolism and PPAR signaling in at least three out of the sixteen tumor types. We merged RNA differentially expressed and survival data from the clinical information file matched by sample ID for each tumor type. The statistical significance of survival differences in the Kaplan-Meier analysis was assessed using the Log-rank test and splitting the tumor samples into two groups: low and high expression levels (median value used as the cut-off), as implemented in Survival-Online [30]. For survival clinical features, Log-rank test in univariate Cox regression analysis with proportional hazards model [31] was used to estimate the *p* values comparing quantile intervals using the ‘coxph’ function from package “rms” in R.

## Additional files

Additional file 1: Lipid metabolism pathways. (XLSX 17 kb)

Additional file 2: Gene expression levels with mutation frequency more than 2% and differently expressed in pan-cancer (PDF 333 kb)

Additional file 3: Transcription factors and Targets (XLSX 35 kb)

Additional file 4: The correlation of transcripition factor and target pairs in key lipid metabolism pathways. (XLSX 52 kb)

Additional file 5: The correlation of gene expression and immune cell proportion in key lipid metabolism  pathways. (XLSX 51 kb)

Additional file 6: Candidate lipid metabolism genes. (XLSX 39 kb)

## Abbreviations

19(S)-HETE: 19(S)-hydroxy-eicosatetraenoic acid
BLCA: Bladder cancer
BRCA: Breast cancer
COAD: Colon adenocarcinoma
EETs: Epoxyeicosatrienoic acids
ESCA: Esophageal carcinoma
HETEs: Hydroperoxyeicosatetraenoic acids
HNSC: Squamous cell carcinoma of head and neck
KICH: Kidney chromophobe cell carcinoma
KIRC: Kidney renal clear cell carcinoma
KIRP: Kidney renal papillary cell carcinoma
LIHC: Liver cancer
LUAD: Lung adenocarcinoma
LUSC: Lung squamous cell carcinoma
PRAD: Prostate cancer
STAD: Stomach adenocarcinoma
TCGA: The Cancer Genome Atlas
THCA: Thyroid cancer
UCEC: Uterine corpus endometrial carcinoma
