# Supplementary material for: Investigation of lipid metabolism dysregulation and the effects on immune microenvironments in pan-cancer using multiple omics data
Source: BMC Bioinformatics. 2019 May 1;20(Suppl 7):195. doi: 10.1186/s12859-019-2734-4 (PMC6509864; doi:10.1186/s12859-019-2734-4)
Supplement: Supplementary file 2 — Gene expression levels with mutation frequency more than 2% and differently expressed in pan-cancer (PDF 333 kb) [file 12859_2019_2734_MOESM2_ESM.pdf]

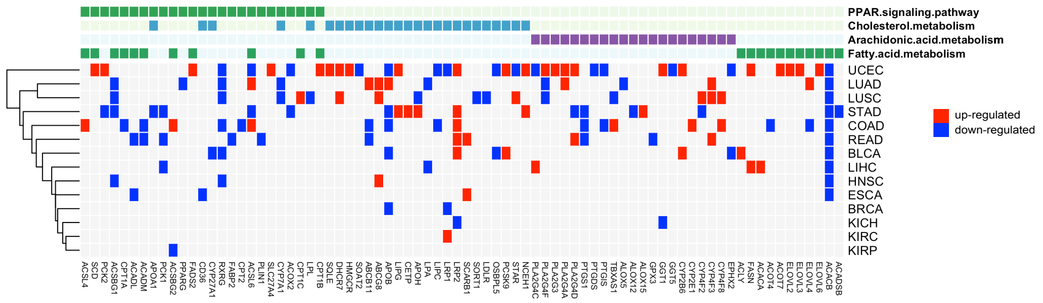

Fig. S1. Gene expression levels with mutation frequency more than 2% and differently expressed in pan-cancer. Genes with up-regulated or low-regulated in four pathways across multiple cancer types are respectively shown in red and blue (|logFC| > 1, FDR < 0.05).
